# Pancoast Tumor: The Role of Magnetic Resonance Imaging

**DOI:** 10.1155/2013/479120

**Published:** 2013-03-31

**Authors:** Guglielmo Manenti, Mario Raguso, Silvia D'Onofrio, Simone Altobelli, Angela Lia Scarano, Erald Vasili, Giovanni Simonetti

**Affiliations:** Department of Diagnostic Imaging, Molecular Imaging, Interventional Radiology and Radiation Therapy, Policlinico Tor Vergata, Viale Oxford 81, 00133 Rome, Italy

## Abstract

We report imaging techniques in the definition of the therapeutic planning of a 65-year-old man with a diagnosis of Pancoast tumor. Computed Tomography has a pivotal role in the assessment of nodes involvement and distant metastasis. Magnetic Resonance allows a detailed study of locoregional extension for its high soft tissue resolution. We particularly highlight the actual importance of Magnetic Resonance Neurography, Diffusion-Weighted Imaging, and Magnetic Resonance Angiography techniques in the assessment of the superior sulcus vascular and nervous structures involvement. Their integrity has been showed in our patient with a complete surgical excision of the lesion.

## 1. Introduction

The superior pulmonary sulcus tumors account for 5% of all cancers [1]. Pancoast tumor often infiltrates parietal pleura, endothoracic fascia and its lymphatic vessels, brachial plexus, intercostal nerves, stellate ganglion, upper ribs, vertebral bodies, and not often subclavian vessels. For the definition of a therapeutic approach, assessment of the integrity of these anatomical structures is of pivotal importance [1, 2].

Second level imaging, which includes techniques like Computed Tomography (CT) and Magnetic Resonance (MR), has actually an important role. 

CT is considered a sensitive imaging technique, especially in the assessment of nodes involvement and distant metastasis.

In the specific evaluation of loco-regional tumor extension (evaluation of brachial plexus, subclavian vessels, parietal pleura, subpleural fat, neurovertebral foramina, and spinal canal involvement), MR provides a high soft tissue resolution. 

Purpose of this report is to show the role of MR in the definition of therapeutic management of apical neoplasms.

## 2. Case Presentation

A 65-year-old man, smoker of 20 cigarettes a day, reported a persistent neck and right shoulder pain. Diagnostic tests for discopathy were negative. A chest X-ray, which showed a radio-opacity in the right lung apex, prompted a diagnosis of Pancoast tumor. A Positron Emission Tomography-Computed Tomography (PET-CT) (*Discovery ST, General Electrics, Medical System, Germany*) examination was performed. The fused PET-CT images were made available on a postprocessing workstation (*Advantage-Windows 4.4, General Electrics, Medical System*), and the diagnosis was confirmed, with the indication for a surgical approach by excluding the presence of metastasis.

The integrity of anatomical structures of the superior sulcus was confirmed with a dedicated study by using the MR. MR examination was performed on a 3.0 T system (*Achieva, Philips, The Netherlands*) with the body coil. MR protocol included

(i)  T1 weighted (T1W) Turbo Spin Echo (TSE) 4 mm images on the axial plane with repetition time (TR) = 400 ms, echo time (TE) = 9.2 ms, flip angle (FA) = 90°, and acquisition matrix (MA) = 512 × 512;
(ii)   T2 weighted (T2W) TSE 4 mm images with a fat-signal suppression on the coronal (TR = 6294 ms, TE = 80 ms, FA = 90°, and MA = 512 × 512) and axial (TR = 7003 ms, TE = 80 ms, FA = 90°, and MA = 512 × 512) planes;
(iii)   T2W TSE 4 mm images on the axial (TR = 6646 ms, TE = 80 ms, FA = 90°, and MA = 512 × 512) and sagittal (TR = 3988 ms, TE = 80 ms, FA = 90°, and MA = 512 × 512) planes;
 (iv)  “Short Tau Inversion Recovery” (STIR) 1,5 mm images (TR = 17000 ms, TE = 60 ms, inversion time (TI) = 200 ms, MA = 384 × 384, and FA = 90°);
(v)   diffusion weighted images with background body signal suppression (DWIBS) 4 mm images on the axial plane for the study of the brachial plexus (TR = 22000 ms, TE = 60 ms; TI = 200 ms, MA = 160 × 160, and FA = 90°);
(vi)   postcontrast agent (Gadolinium-DTPA) 3D high resolution MR angiography (MRA) on the coronal plane (TR = 5.5 ms, TE = 1.5 ms, FA = 30°, and MA = 512 × 512);
(vii)   post-Gadolinium T1W TSE 4 mm images on the coronal and axial planes (TR = 400 ms, TE = 9.2 ms, FA = 90°, and MA = 512 × 512).

**Figure 1:**
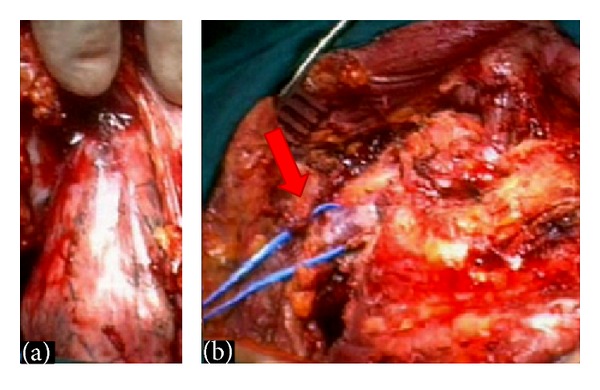
Intraoperative view of a superior sulcus tumor: (a) The nervous and vascular structures lie above the tumor mass. With a correct surgical approach a posterior chest wall attached to round-shaped lesion was isolated from surrounding soft tissues. (b) The lesion was entirely removed without injury of the nervous and vascular structures. A full red arrow shows the integrity of the subclavian vein.

A surgical excision of the lesion was finally obtained with excellent results (Figure 1).

## 3. Discussion

The superior pulmonary sulcus is a groove delimited by subclavian artery which passes over the lung at this level.

Local anatomy description was due to pathologist H.K. Pancoast who reported the first case tumor in a series of publications between 1924 and 1932 [2]. 

According to the loco-regional extension of the neoplasm, there is a wide range of clinical presentations. Shoulder and neck pain is often present. 

There are sensorial and motor disorders of neck and arms in the case of intervertebral foramens or brachial plexus implication [2]. The involvement of the inferior portion of the brachial plexus (exactly the eight cervical nerve and the first and second thoracic trunk) is the reason of the Pancoast syndrome: neck and medial arm pain, paresthesias, weakness, and late muscle dystrophy are the consequences. 

A cervical radiculopathy or a cuff-rotator lesion is a common clinical bias [3]. 

Superior vena cava syndrome or recurrent nerve palsy may occur as late complications of the disease progression [4].

Invasion of the stellate ganglion is the reason of the Horner syndrome. Typical signs are miosis, ptosis, enophthalmos, and anhidrosis of the affected side [5]. 

Because of the wide range of clinical presentations, the role of imaging is pivotal in the diagnosis and evaluation of the therapeutic approach.

### 3.1. Role of Imaging

In the past the chest plain radiography had a predominant role in the diagnosis of an apical tumor, which often was seen as a radio-opacity in the superior sulcus of the lung [5]. 

CT is considered a standardized technique in the evaluation of the loco-regional extension but especially of nodes involvement and distant metastasis [6]. 

Exclusion criteria for surgical resection are the invasion of the brachial plexus at a level above the T1 nerve, the destruction of more than 50% of the vertebral bodies, TNM N2 (mediastinal) or N3 (supraclavicular contralateral) node involvement, and distant metastases [7]. CT is the gold standard imaging technique, especially for the evaluation of the bone erosion (vertebral body and rib involvement).

Lymph-node staging is based on morphological criteria. The radiologist targets his attention on lymph nodes with a size > 1 cm. Thin layer multislice CT images are more helpful in this aim [6].

**Figure 2:**
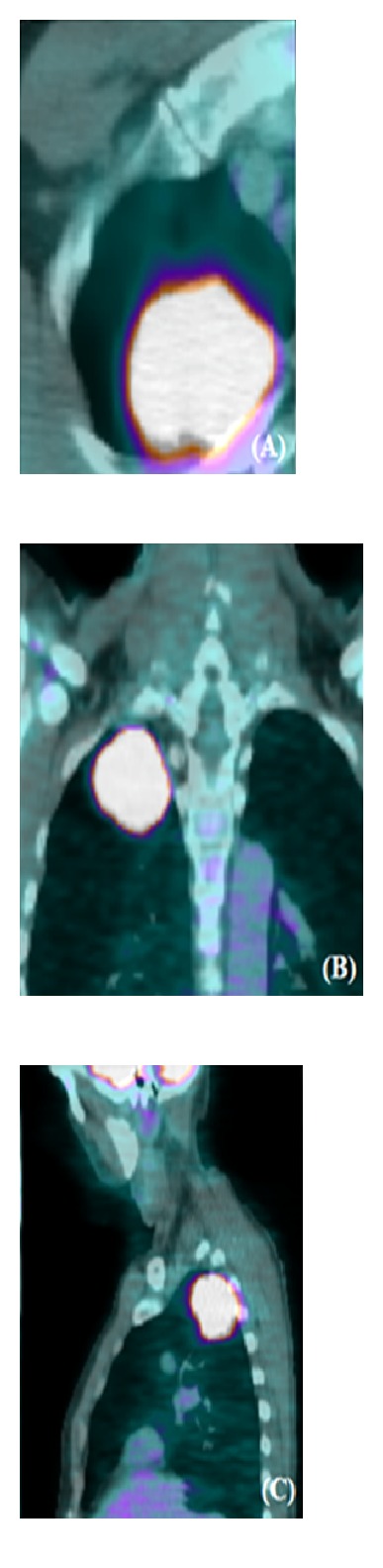
In PET-CT a high 18F-fluorodeoxyiglucose uptake confirms the neoplastic nature of a superior sulcus lesion. CT also allows a morphological study thanks to the possibility of multiplanar reformations on the axial (A), coronal (B), and sagittal planes (C).

Actually there is an increasing use of PET-CT for detection of lesions with a high metabolic rate (Figure 2). PET-CT joins the high spatial resolution with the functional date, by identifying tracer uptake as small as 5 mm.

### 3.2. MR Imaging in the Study of Pancoast Tumor

For evaluation of loco-regional extension (particularly brachial plexus, subclavian vessels, parietal pleura, subpleural fat, neurovertebral foramina, and spinal canal), MR provides a higher soft tissue resolution compared to CT [5, 7]. 

By using surface coils for anatomical definition, a study of the lung apical structures is based on thin layer (3-4 mm) SE or TSE T1W and T2W images protocol [8, 9].

**Figure 3:**
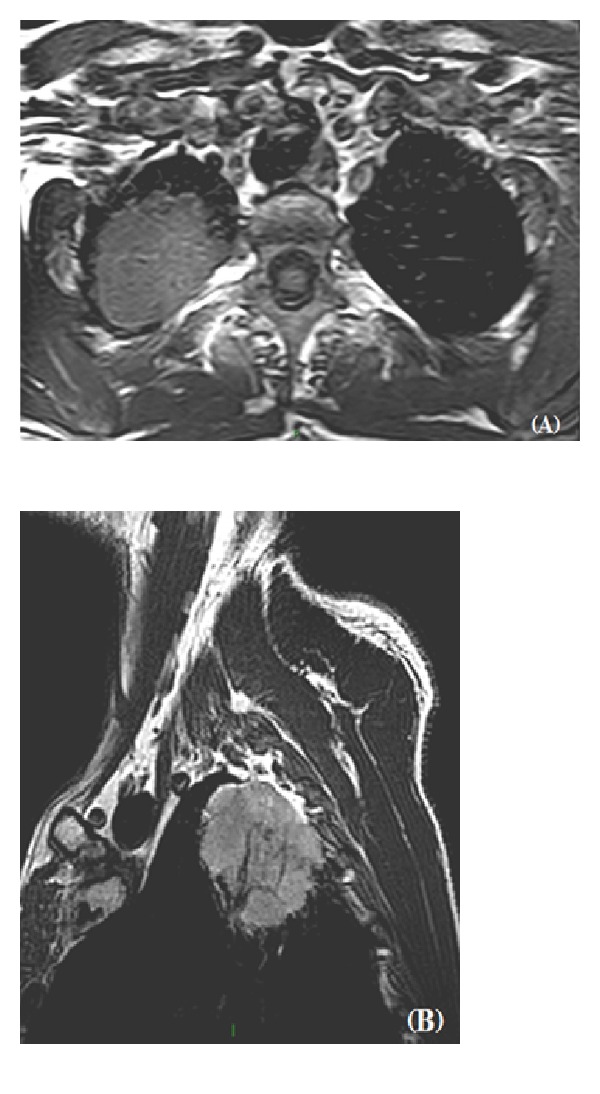
MR technique has a pivotal role in the assessment of the integrity of superior sulcus. The higher soft tissue resolution and the possibility to directly obtain images on axial (A) and sagittal (B) planes may help define the boundaries of an apical neoplasm.

T1W images have high anatomical resolution (Figure 3). Sagittal T1W sequences provide the most detailed anatomic information and should be performed first in order to visualize the relationship of the subclavian vessels with other anatomical components [7]. 

**Figure 4:**
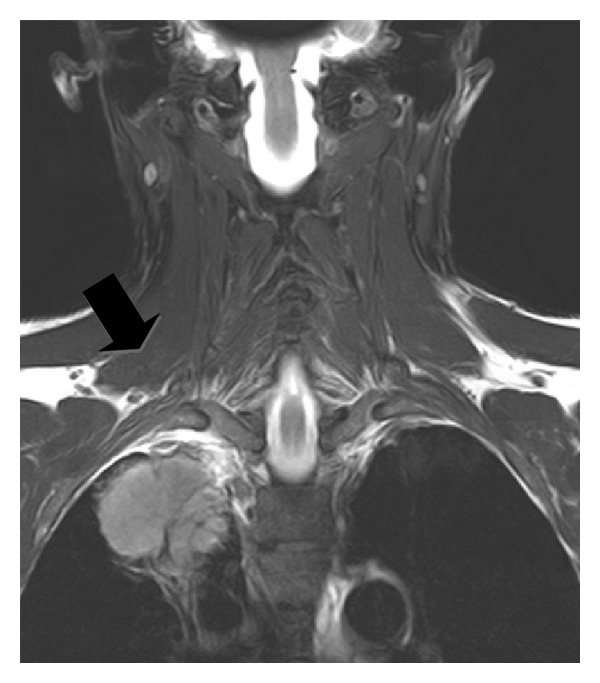
This TSE T2W image on the coronal plane shows the integrity of the anatomical structures of the superior sulcus (full black arrow).

Subclavian vessels have a horizontal course and a low intensity of signal in T1W images [10]. MR imaging on the coronal plane is more specific for the evaluation of their involvement [11]. On the coronal T2W images, the inter-scalenes fat has a triangular morphology. Its interruption in MR images excludes surgical resection (Figure 4) [12]. 

**Figure 5:**
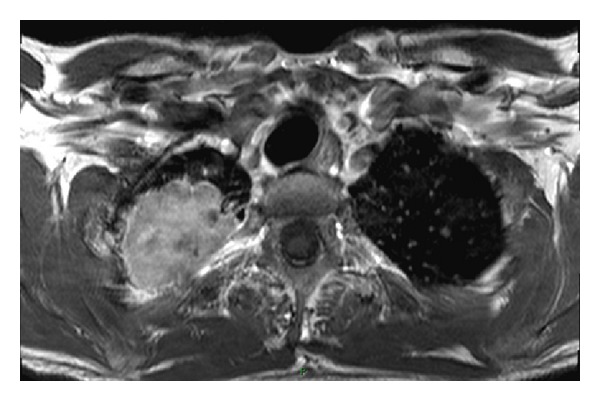
Post-Gadolinium T1W TSE axial image. The intravenous injection of Gadolinium is helpful in the assessment of a vascular invasion and may also help distinguish between posttreatment fibrosis and recurrence of the neoplasm.

T1W MR images on the three planes, after the intravenous injection of Gadolinium DTPA, may help to define the boundaries of an apical lesion (Figure 5) [8]. 

Contrast medium is helpful for the assessment of a vascular invasion [7]. It may also help to distinguish between posttreatment fibrosis and recurrence of the neoplasm. 

The limit of the conventional T1W and T2W sequences is the lack of three-dimensional imaging, which represents nerve roots throughout their length [10].

The study of the superior sulcus can be completed by using T2W sequences with fat-signal suppression.

The endoneural fluid of each nerve has a strong signal intensity in T2W images. Use of T2W sequences with fat-signal suppression is mandatory in the study of the brachial plexus [9]. 

**Figure 6:**
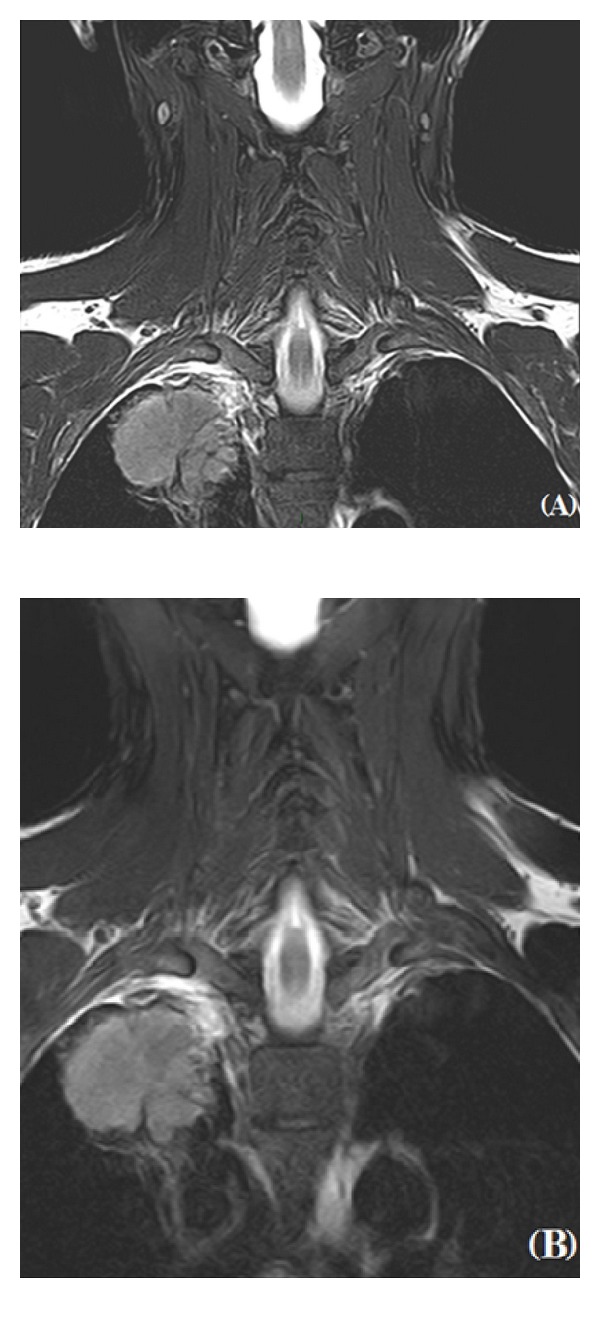
The use of TSE T2W sequences with a fat-signal suppression is mandatory in the study of the brachial plexus. Images on the coronal plane (A) provide the possibility to study the entire superior sulcus and to evaluate its integrity in case of Pancoast tumor. The opportunity to process digital images also allows obtaining a magnification for a better assessment of the brachial plexus and a vascular invasion (B).

There are two main methods to make hypointense the signal of fat. The use of a prefrequency selective impulse for fat-signal suppression has the disadvantage of an inhomogeneous fat suppression, due to the different anatomical structures of the superior sulcus (Figure 6).

**Figure 7:**
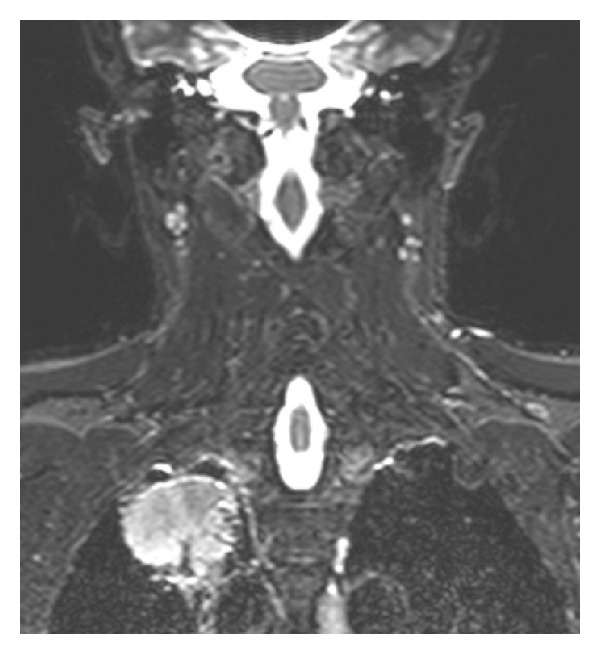
In this STIR image on the coronal plane it is showed a round-shaped lesion without an invasion of the superior sulcus. STIR sequences provide a better signal homogeneity, but they have a lower SNR.

STIR imaging is a second technique to obtain a fat-signal suppression. It has the characteristics of T2W images with saturation of the fat-signal. STIR sequences provide a better homogeneity, but they have a lower Signal-Noise Ratio (SNR) than T2W imaging with pre-frequency for a fat suppression (Figure 7) [9]. Nerve roots appear hyperintense with fat-signal hypointensity at STIR imaging. This technique is conventionally reported as MR neurography [13]. 

Imaging on the sagittal plane is preferred for a cross-sectional evaluation of the brachial plexus, while axial and coronal images provide a global vision of the entire trunks and their relationship with other anatomical components of the superior sulcus. STIR sequences offer also the best contrast between tumor and oedema for assessment of bones, nodes and soft tissues involvement [13, 14]. 

**Figure 8:**
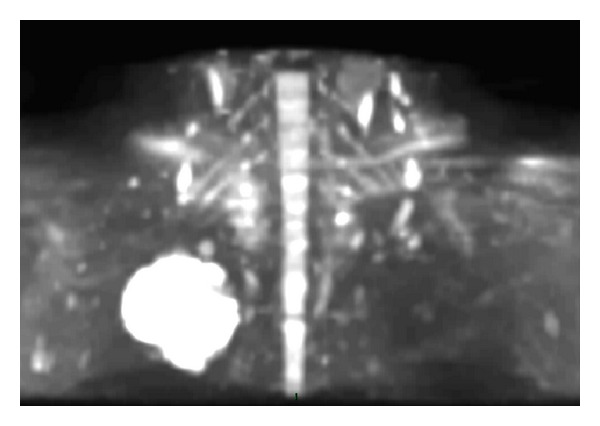
MIP reformatted DW image on the coronal plane which shows the integrity of the brachial plexus. DW imaging makes the radiologist able to selectively identify nervous structures, which have a strong signal intensity and are then clearly visible between the other components of the superior sulcus (which conversely are hypointense). Postprocessing MIP technique allows assessment of the entire brachial plexus.

Diffusion weighted (DW) imaging provides the possibility to selectively identify nodes involvement and nervous structures themselves. Nerve roots have a strong signal intensity, which makes it clearly visible between the other components of the superior sulcus (which conversely are hypointense) [13]. Postprocessing techniques, such as Maximum Intensity Projection (MIP), on coronal DW images allow assessment of the entire brachial plexus (Figure 8) [10].

Involvement of subclavian vessels is demonstrated by 3D MRA [15] with the intravenous injection of Gadolinium DTPA (Contrast Enhanced Magnetic Resonance Angiography technique CEMRA), by acquisition of multiple overlapping 3D volumes of small thickness in a sequential mode in order to obtain a high sensitivity and less motion artefacts. Diagnostic confidence is finally increased by MIP reformatted images [13]. 

## 4. Conclusions

MR imaging has a pivotal role for staging and therapeutic management in patients with Pancoast tumor. MR neurography, DW imaging, and MR angiography are actually mandatory imaging techniques in all patients with superior pulmonary sulcus tumors.
